# Obtaining and Characterizing the Osmium Nanoparticles/*n*–Decanol Bulk Membrane Used for the *p*–Nitrophenol Reduction and Separation System

**DOI:** 10.3390/membranes12101024

**Published:** 2022-10-21

**Authors:** Aurelia Cristina Nechifor, Alexandru Goran, Szidonia-Katalin Tanczos, Florentina Mihaela Păncescu, Ovidiu-Cristian Oprea, Alexandra Raluca Grosu, Cristian Matei, Vlad-Alexandru Grosu, Bogdan Ștefan Vasile, Paul Constantin Albu

**Affiliations:** 1 Analytical Chemistry and Environmental Engineering Department, University Politehnica of Bucharest, 011061 Bucharest, Romania; 2 Department of Bioengineering, University Sapientia of Miercurea-Ciuc, 500104 Miercurea-Ciuc, Romania; 3 Department of Inorganic Chemistry, Physical Chemistry and Electrochemistry, University Politehnica of Bucharest, 011061 Bucharest, Romania; 4 Department of Electronic Technology and Reliability, Faculty of Electronics, Telecommunications and Information Technology, University Politehnica of Bucharest, 061071 Bucharest, Romania; 5 National Research Center for Micro and Nanomaterials, Politehnica University of Bucharest, 011061 Bucharest, Romania; 6 Radioisotopes and Radiation Metrology Department (DRMR), IFIN Horia Hulubei, 023465 Măgurele, Romania

**Keywords:** osmium nanoparticles, liquid membranes, *n*–decanol, 1–undecenoic acid, dispersion bulk liquid membrane system, *p*–nitrophenol reduction, *p*–aminophenol transport

## Abstract

Liquid membranes based on nanoparticles follow a continuous development, both from obtaining methods and characterization of techniques points of view. Lately, osmium nanoparticles have been deposited either on flat membranes, with the aim of initiating some reaction processes, or on hollow fiber membranes, with the aim of increasing the contact surface with the phases of the membrane system. This paper presents the obtainment and characterization of a liquid membrane based on osmium nanoparticles (Os–NP) dispersed in *n*decanol (*n*Dol) for the realization of a membrane system with a large contact surface between the phases, but without using a liquid membrane support. The dispersion of osmium nanoparticles in *n*-decanol is carried out by the method of reducing osmium tetroxide with 1–undecenoic acid (UDA). The resulting membrane was characterized by transmission electron microscopy (TEM), scanning electron microscopy (SEM), energy-dispersive spectroscopy analysis (EDAX), thermoanalysis (TG, DSC), Fourier transform infra-red (FTIR) spectroscopy and dynamic light scattering (DLS). In order to increase the mass transfer surface, a design for the membrane system was realized with the dispersion of the membrane through the receiving phase and the dispersion of the source phase through the membrane (DBLM-dispersion bulk liquid membrane). The process performance was tested for the reduction of *p*–nitrophenol (pNP) from the source phase, using sodium tetra-borohydride (NaBH_4_), to *p*–aminophenol (pAP), which was transported and collected in the receiving phase. The obtained results show that membranes based on the dispersion of osmium nanoparticles in *n*–decanol can be used with an efficiency of over 90% for the reduction of *p*–nitrophenol and the separation of *p*–aminophenol.

## 1. Introduction

**Figure 1:**
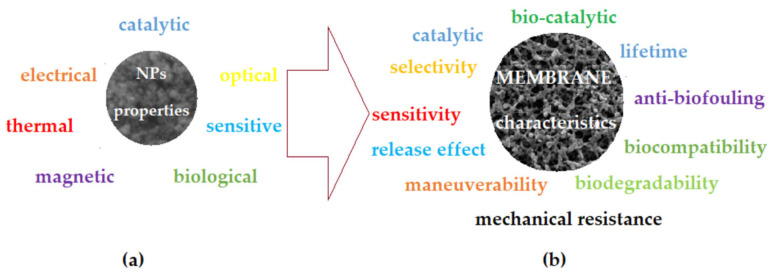
Schematic presentation of the involvement of the properties of nanoparticles (**a**), and the properties needed to achieve the characteristics of membranes (**b**).

Enormous advances in nanomaterials and nanotechnology have made a significant impact on the field of membranes and membrane processes [1,2]. Membranes are themselves, in many cases, highly selective nanostructures used in the separation, concentration and purification of various complex systems [3]. It was expected that the field of membranes would benefit from the special properties of nanomaterials [4], but especially those of natural or synthetic nanoparticles [5], whether these be organic, inorganic or composite [6]. Among the inorganic nanoparticles, the metallic ones are of particular interest for their excellent properties (Figure 1a): mechanical, thermal, electrical, magnetic, catalytic, sensitive and/or biological [7,8,9,10]. Of course, the process characteristics and performances of the membranes were thus improved in terms of flow, mechanical resistance and lifetime, selectivity and sensitivity, and biocompatibility and biodegradability [11,12,13].

Among the metallic nanoparticles, those based on osmium have been relatively little studied [14,15,16], with one of the reasons being toxicity [17]. In reality, osmium is an inert metal with remarkable potential mechanical, thermal, electrical and catalytic properties, and its toxicity is related to osmium tetroxide that appears on the metal surface from contact with air [18]. However, if osmium nanoparticles are embedded in a membrane material, it can benefit from the catalytic properties marked by specificity and regioselectivity [19,20], which are very difficult to obtain by other means.

On the other hand, the fact that osmium has a high market value requires it to be recovered from various sources of osmium tetroxide [21]. 

As an industrial residue of copper metallurgy, that is also widely used on a large scale in microscopy laboratories in the field of polymers or biological materials, osmium tetroxide can become a raw material for the generation of nanoparticles used to make membranes [22].

The reduction of osmium tetroxide is most often performed with molecular hydrogen (Equation (1)), but many reducing agents, especially organic ones, can change the tetroxide metallic osmium (Equation (2)):

OsO_4_ + 4 H_2_ → Os + 4 H_2_O (1)

OsO_4_ + Red → Os + Ox (2)

In recent years, osmium nanoparticles have been deposited either on flat membranes with the aim of initiating reaction processes [14], or on hollow fiber membranes with the aim of increasing the contact surface between the phases of the membrane system [15,16].

This paper presents the obtainment and characterization of a liquid membrane based on osmium nanoparticles (Ox–NP) dispersed in *n*-decanol (*n*Dol) for the realization of a membrane system with a large contact surface between the phases, but without using any support for the liquid membrane. The obtained membrane was morphologically and structurally characterized and the process performances in the reduction of *p*–nitrophenol are presented (*p*NP).

## 2. Materials and Methods

### 2.1. Materials

The materials used in the presented work were used without supplementary purification. The following materials were purchased from Merck (*Merck KGaA*, Darmstadt, Germany): osmium tetroxide (OsO_4_), sodium borohydride (NaBH_4_, 96%), sodium hydroxide, and hydrochloric acid. The rest were purchased from Sigma-Aldrich (*Merck KGaA*, Darmstadt, Germany): *p–*nitrophenol, tert-butanol, *i–*propanol, *n*–decyl alcohol (*n*–decanol) (molar mass: 158.28 g/mol; density: 830 kg/m^3^; solubility in water: 0.037 mg/L), 10–undecylenic acid (1–undecenoic acid; molar mass: 184.28 g/mol, density: 912 kg/m^3^; solubility in water: 0.074 mg/mL). 

The purified water characterized by 18.2 μS/cm conductivity was obtained with an RO Millipore system (MilliQ^®^ Direct 8 RO Water Purification System, Merck KGaA, Darmstadt, Germany).

### 2.2. Procedures

#### 2.2.1. Preparation of Osmium Dispersion in *n*–Decanol

**Figure 2:**
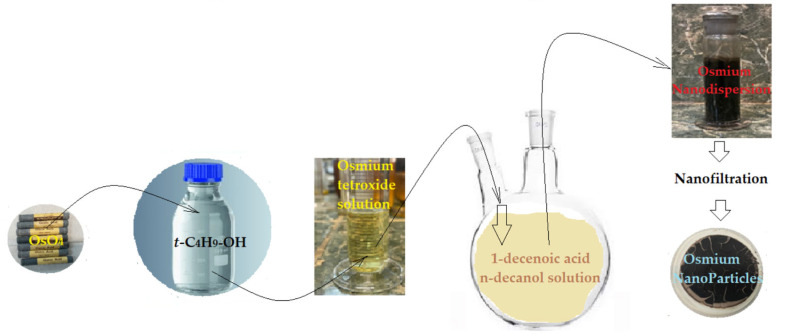
Schematic presentation of obtaining the osmium nanoparticles and their dispersion in *n*-decanol.

The process of obtaining osmium nanoparticles intended for the preparation of membranes is well-known [15,16] and involves the reduction of an osmium tetroxide solution in tert–butanol with 1–undecenoic acid (UDA). In this work, the predetermined amount of osmium tetroxide solution (10 g/L) was injected into 1 L of 10^−3^ mol/L of 1–undecenoic acid solution in *n*–decanol (Figure 2).

The reduction reaction of osmium tetroxide is instantaneous. The resulting nanodispersion can be used in the membrane process but, if necessary, it can also be separated by nanofiltration, when osmium nanoparticles are obtained. The advantage of this preparation technique is that the obtained nanoparticles are suspended in *n*-decanol, with the nanodispersion itself being non-sedimentable. Nanodispersions were prepared with concentrations of 0.5, 1.0, and 1.5 g/L of osmium nanoparticles in *n*–decanol.

#### 2.2.2. Membrane System with Dispersion

**Figure 3:**
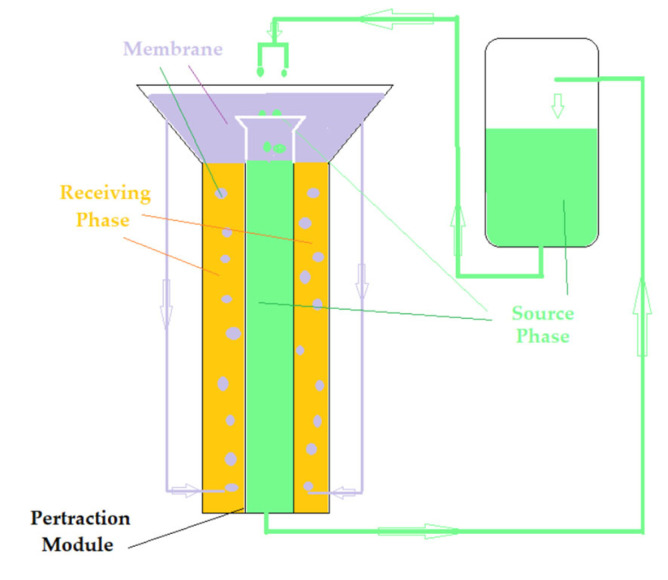
Schematic of the pertraction installation, which uses a module with phases in dispersion.

The design of the pertraction ensemble was conceived to achieve the stirring of the phases, and to increase the contact surface between them without mechanical means inside (Figure 3).

In the transport installation (Figure 3), the receiving phase, with a volume of 1 L, is stationary, the source phase, with a volume of 10 L, is recirculated with a flow rate of 20–120 mL/min using a peristaltic pump, and the 500 mL membrane is recirculated at a flow rate of 5–50 mL/min. *p*–nitrophenol concentrations are determined at predetermined time intervals.

#### 2.2.3. Reduction and Transport Experiments

The study of the reduction and transport of the target chemical species (*p*–nitrophenol and *p*–aminophenol) was performed using an installation of our own design (Figure 3). The target chemical species were dissolved in pure water to form two stock solutions with a concentration of 10^−3^ mol/L.

The source phase contains *p*-nitrophenol and sodium borohydride in concentrations of 10^−4^–10^−2^ mol/L, and the receiving phase contains a solution of hydrochloric acid with a concentration of 10^−4^–10^−1^ mol/L.

The concentrations of the studied chemical species and their pH were obtained using sodium borohydride, sodium hydroxide, and hydrochloric acid in the source or receiving phase. The adjustment was completed with freshly prepared sodium hydroxide solution (10^−1^ mol/L).

### 2.3. Equipment

The scanning electron microscopy (SEM) and high-resolution scanning electron microscopy (HR-SEM) studies were performed on a Hitachi S4500 system (Hitachi High-Technologies Europe GmbH, Krefeld, Germany) [23]. 

The transmission electron microscopy (TEM) studies were recorded with a high-resolution 80–200 kV Titan THEMIS transmission microscope (Thermo Fisher Scientific, Hillsboro, OR, USA) equipped with an Image Corrector and EDXS detector in the column. The microscope was operated at 200 kV in transmission mode. The HAADF (high annular dark field) images were obtained using STEM mode [24].

Thermal analysis, TG-DSC (thermo-gravimetric and differential scanning calorimetry), was performed with an STA 449C F3 apparatus from Netzsch (Netzsch-Gerätebau GmbH, Selb, Germany), between 20 and 350 °C, in a dynamic (50 mL/min) N_2_ atmosphere. The evolved gases were analyzed with a FTIR Tensor 27 from Bruker (Bruker Co., Ettlingen, Germany), equipped with a thermostatic gas cell.

The thermal analysis was run in a nitrogen atmosphere at a heating rate of 10 °C/min, from room temperature (RT = 25 °C) up to 900 °C [25].

The study conditions for each method of analysis were as follows:

1. Dynamic light scattering (DLS) analysis: granulometry equipment: Coulter N4 Plus (He–Ne laser, 632.8 nm); analysis range: 3–3000 nm; detection angle: 10.7°; RT analysis temperature: 23 °C ±1; stabilization time at RT: 5 min; analysis time: auto; data collection time: 5 min × 10 (repetitions); ultrasound time (US): 5 min (20 kHz, RT); rest time after US: ~24 h; dispersion medium (solvent): *i*–propanol; sample dilution: ~1:500.
2. Size distribution processor (SDP) analysis: ultrasound time (US): 5 min (20 kHz, RT); rest time after US: ~24 h [26].

The UV–Vis analyses of the aqueous nitrophenol solutions were performed using a Spectrometer CamSpec M550 (Spectronic CamSpec Ltd., Leeds, UK) [27].

The UV–Vis studies on the nanoparticle samples were performed with dual-beam UV equipment known as Varian Cary 50 (Agilent Technologies Inc., Santa Clara, CA, US), at a resolution of 1 nm, spectral bandwidth of 1.5 nm, and 300 nm/s scan rate. The UV–Vis spectra of the samples were recorded for wavelengths from 200 to 800 nm, at room temperature, using 10 mm quartz cells [28].

Monitoring the concentration of the chemical species in the membrane system phases was performed by ultraviolet and visible spectrometry (UV–Vis) for *p*–nitrophenol and *n*–alcohol [29,30].

The extraction efficiency (EE %) or conversion (*η*%) for *p*–nitrophenol to *p*–aminophenol was calculated as follows [31,32,33], based on the solution concentration:

(3) $$EE\left(\%\right)\;or\;\eta \left(\%\right)=\frac{\left(c_{0}-c_{f}\right)}{c_{0}}\cdot 100$$

with *c_f_* being the final concentration of the solute (*p*–nitrophenol), and *c*_0_ being the initial concentration of solute (*p*–nitrophenol).

The same extraction efficiency or conversion (*η*%) can also be obtained based directly upon the absorbance of the considered solutions (*p–*nitrophenol) [34,35,36], as in:

(4) $$EE\left(\%\right)\;or\;\eta \left(\%\right)=\frac{\left(A_{0}-A_{s}\right)}{A_{0}}\cdot 100$$

with *A*_0_ being the initial absorbance of the sample solution, and *A_s_* being the current absorbance of the sample.

## 3. Results and Discussions

The realization of a liquid membrane based on osmium nanoparticles (Os–NP) dispersed in a biodegradable solvent is an objective that would lead to the development of reaction processes (both reduction and oxidation) applicable to ecological and greening technologies.

This paper presents the obtainment and characterization of a liquid membrane based on osmium nanoparticles obtained directly in *n*-decanol (*n*Dol), which is the basic solvent of the liquid membrane.

The osmium reduction method is well known [16] and is based on the reduction of osmium tetroxide with 1–undecenoic acid (UDA).

Process performances were determined using the reduction reaction of *p*–nitrophenol (*p*NP), with hydrogen generated in situ using sodium borohydride (NaBH_4_), to *p*–aminophenol (*p*AP).

The choice of *p*–nitrophenol as the target substance is justified both by the practical importance of the reaction, and by the fact that there are multiple and excellent studies that thus allow the comparative evaluation of the results [37,38,39,40].

Of course, the realization of an Os–NP/*n*Dol membrane can ensure the study of mild oxidation and reduction reactions for various other species of interest in environmental protection, biosynthesis and bioseparation, and is being expected to exploit the regio- and stereo-specificity of osmium [41,42,43,44].

The membrane system, in which nanodispersion is used to study the reduction reaction from the source phase, but also for the transport of the reaction product in the receiving phase, is designed to increase the contact surface between the phases.

### 3.1. Morpho-Structural Characterization of the Osmium/n–Decanol Nanoparticle Membrane (Os–NP/nDol)

To characterize the osmium nanoparticles/*n*–decanol (Os–NP/*n*Dol) nanodispersion, transmission electron microscopy (TEM), scanning electron microscopy (SEM), thermoanalysis (TG, DSC) and dynamic light scattering (DLS) were used which, through their complementarity, provide the basic characteristics [45] of the membrane material.

#### 3.1.1. Characterization Using Transmission Electron Microscopy (TEM)

The sample was prepared by taking 100 µL of solution containing Os nanoparticles diluted in 1 mL of pure ethanol. After this, 10 µL of the suspension was put onto a 400-mesh holey carbon-coated copper grid at room temperature. The samples were analyzed using a high-resolution 80–200 kV Titan THEMIS transmission microscope (Thermo Fisher Scientific) equipped with an Image Corrector and EDXS detector in the column. The microscope was operated at 200 kV in transmission mode. The HAADF (high annular dark field) images were obtained using the STEM mode.

**Figure 4:**
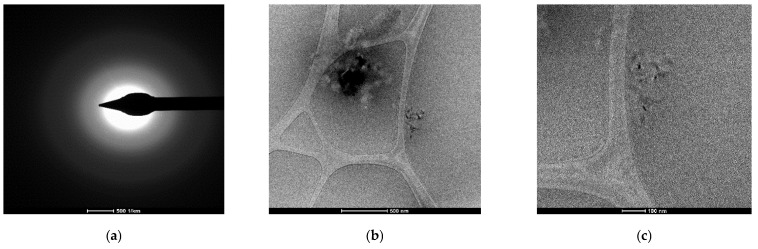

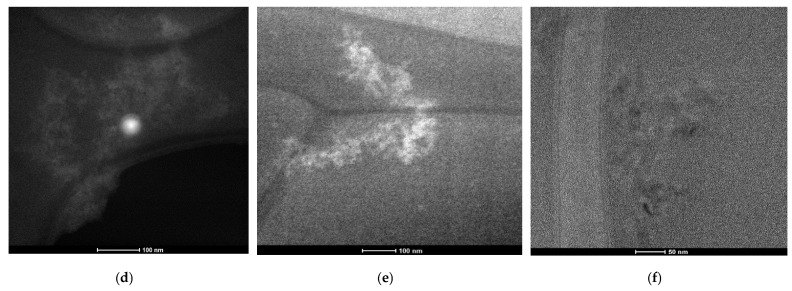
Transmission electron microscopy (TEM) images of the membrane nanodispersions before use in the process. (**a**) Selected area diffraction pattern and images at selected magnitudes of: (**b**) 500 nm; and (**c**) 100 nm. (**d**) Highlight of the exposure point at 100 nm; (**e**) The set of nanoparticles at 100 nm; (**f**) detail of the set of nanoparticles at 50 nm.

**Figure 5:**
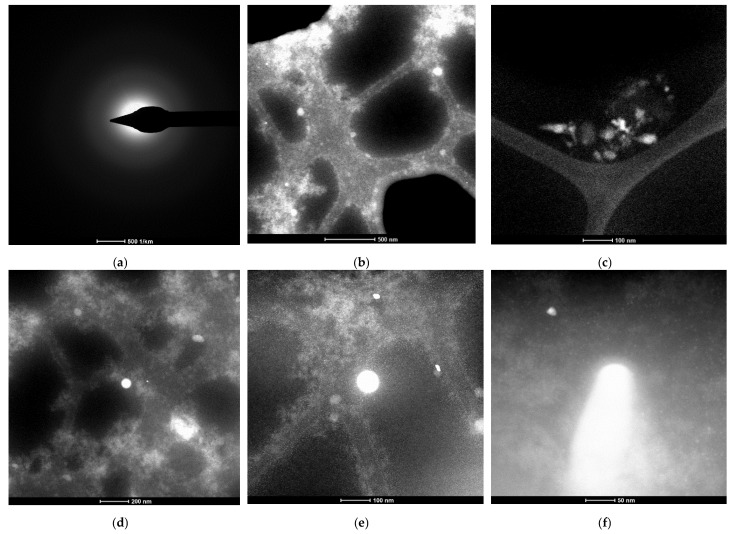
Transmission electron microscopy (TEM) images of membrane nanodispersions after use in the process. (**a**) Selected area diffraction pattern and images at selected magnitudes of: (**b**) 500 nm; and (**c**) 100 nm; (**d**) Highlight the exposure points at 200 nm; and details at: (**e**) 100 nm; and (**f**) 50 nm.

Through the use of transmission electron microscopy (TEM), the membrane nano-dispersions were explored both before (Figure 4) and after they were used in the *p*–nitrophenol reduction process (Figure 5). The insufficient resolution was caused by the sensitivity of osmium nanoparticles coated with *n*–decyl alcohol and 1–undecenoic acid when focusing the electronic beam, especially at high energies. This sensitivity was realized in the instant vaporization of the sample in the place we were examining (Figure 4d and Figure 5d–f light points). The most specific case is highlighted in Figure 5f, in which, in the central light created by the combustion of osmium, nanoparticles below 10 nm can be seen in the background.

#### 3.1.2. Characterization Using Scanning Electron Microscopy (SEM)

The liquid membrane samples for scanning electron microscopy (SEM) were deposited with a micropipette on an aluminum support, evaporated under high vacuum, and a gold layer was deposited to protect the nanomaterial (Os–NP/*n*Dol). 

Although, as a precaution, the electrical and thermal protection of the material was ensured using the method of preparing the samples at high energies. The membrane material vaporized, most likely from the formation of osmium tetroxide from the nanoparticles covered with an organic layer containing oxygen (both *n*–decyl alcohol and 1–undecenoic acid).

**Figure 6:**
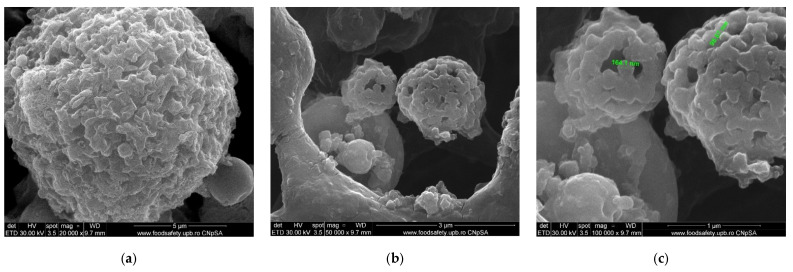
Scanning electron microscopy (SEM) images of the membrane nanodispersion, at magnitudes of: (**a**) ×20,000; (**b**) ×50,000; and (**c**) ×100,000.

However, relevant images of the nanoparticles as aggregates were obtained (Figure 6a,b) and their size was highlighted (Figure 6c), but their intimate structure could not be explored.

**Figure 7:**
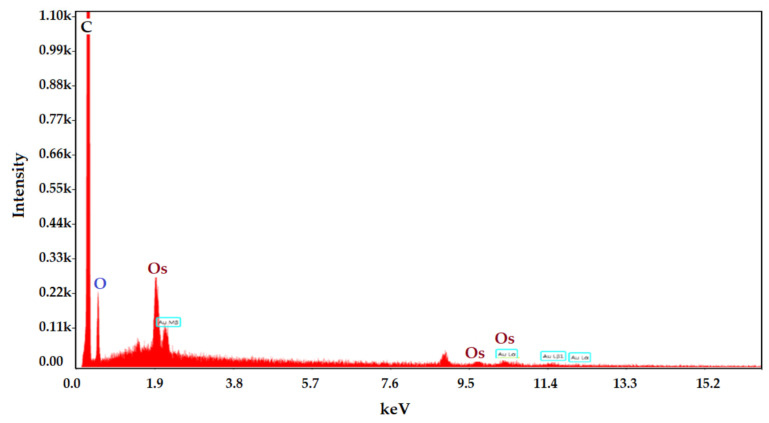
The surface spectrum of the membrane nanodispersion sample obtained by energy-dispersive spectroscopy analysis (EDAX).

The coating of osmium nanoparticles with *n*–decyl alcohol and 1–undecenoic acid was confirmed by examining the surface of the sample in energy-dispersive spectroscopy analysis (EDAX) (Figure 7).

#### 3.1.3. Thermogravimetry and Differential Scanning Calorimetry (TG–DSC) Characterization

Thermal analysis, thermogravimetry and differential scanning calorimetry (TG–DSC), were performed using the STA 449C F3 apparatus from Netzsch (Netzsch-Gerätebau GmbH, Selb, Germany) at temperatures between 20 and 350 °C in a dynamic (50 mL/min) N_2_ atmosphere. The evolved gases were analyzed with an FTIR Tensor 27 from Bruker (Bruker Co., Ettlingen, Germany), equipped with a thermostatic gas cell.

**Figure 8:**
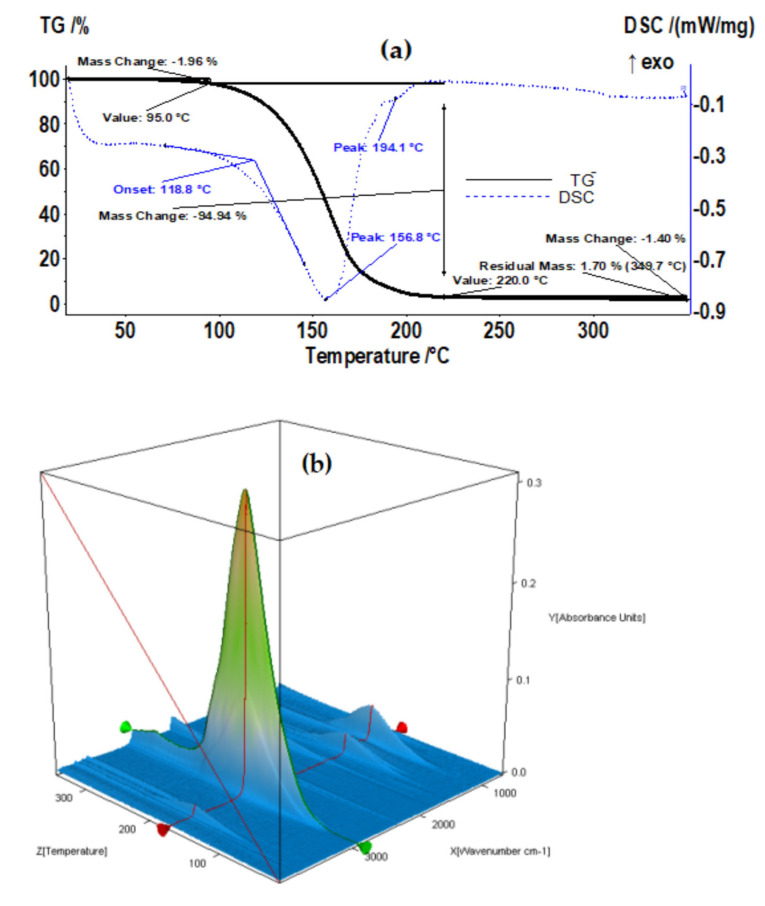

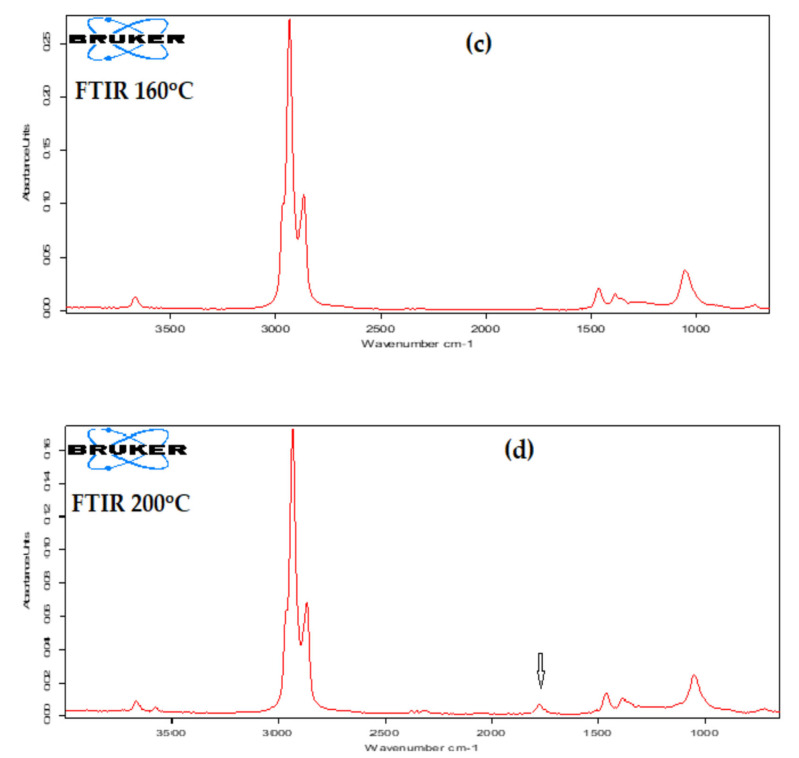
The thermal (**a**) and spectral (**b**–**d**) characteristics of membrane nanodispersion.

The studied sample (Figure 8a) started to lose the liquid part at temperatures over 100 °C, with the evaporation between 95 and 220 °C representing 94.94% of the initial mass. The process was accompanied, on a DSC curve, by an endothermic effect with a minimum at 156.8 °C and a shoulder at 194.1 °C, generated by the mix of the two liquids, *n*–decanol and undecylenic acid. However, in both cases, the boiling points were lower than the values reported by the literature, at 232.9 and 275 °C, respectively. The residual mass was 1.70% and consisted of osmium compounds.

From the Fourier transform infra-red (FTIR) spectrometry (Figure 8b–d) for gases, at 200 °C (Figure 8d), the appearance of a peak at 1721 cm^−1^ was noticeable, corresponding to the C=O bond from the carboxylic group. This indicates, as expected, that undecylenic acid evaporates after *n*–decanol.

#### 3.1.4. Characterization by Dynamic Light Scattering (DLS)

To confirm the characteristics of the membrane nanodispersion, samples from the Os–NP/*n*Dol membrane, taken both before and after the *p*–nitrophenol reduction and transport process, were diluted in *i*–propanol, at a dilution of 1:500 (volumetric).

**Figure 9:**
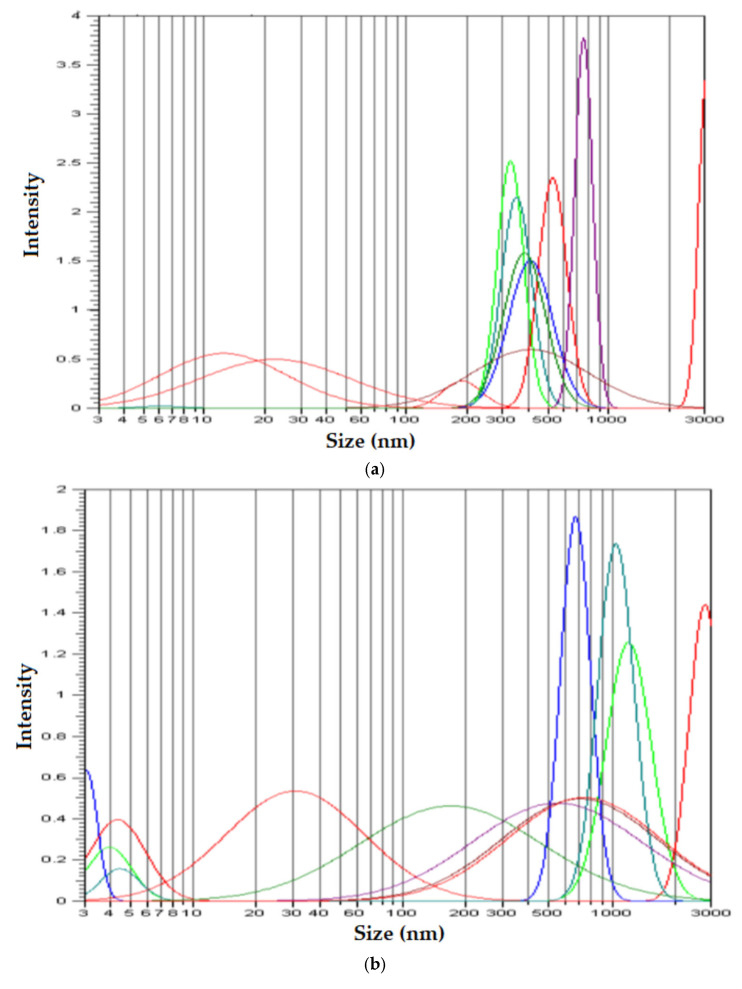

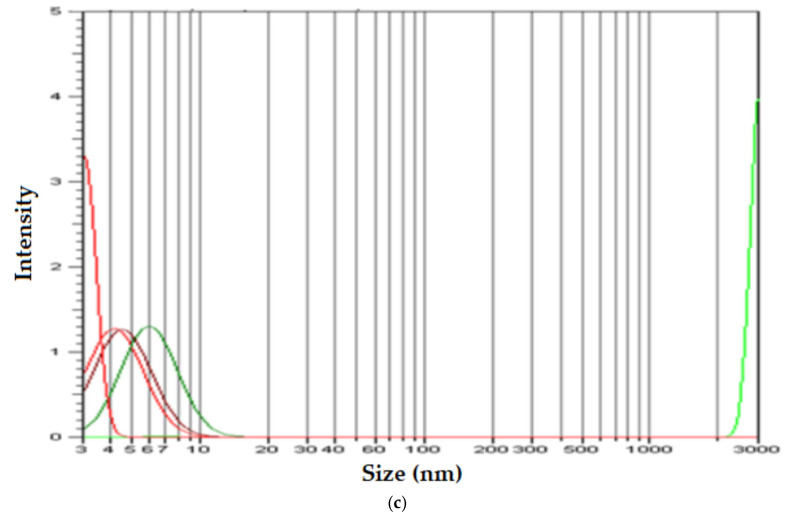
Size distribution of nanospecies in membrane nanodispersion: (**a**) initial nanodispersion; (**b**) nanodispersion after use in the reduction and transport process; (**c**) the initial nanodispersion subjected to ultrasound.

In order to obtain the most accurate information, the measurements were repeated and acquired ten times for each sample (Figure 9).

The results of the samples not subjected to ultrasound (Figure 9a,b) confirmed the results obtained by transmission electron microscopy (TEM) (Figure 4b–d and Figure 5b–e, respectively), which showed that the nanodispersion contains aggregates of sizes between 500 and 1000 nm. The images obtained by scanning electron microscopy (SEM) (Figure 6) clearly showed that the 500–1000 nm area is characterized by the presence of aggregated nanoparticles, both in the initial membrane nanodispersion and in the one after processing.

Because some of the nanoparticles were even less aggregated in assemblies (Figure 9a,b) or even not aggregated (3–100 nm area), we subjected the initial sample to ultrasound. The size distribution of non-aggregated nanoparticles (Figure 9c) showed that the dimension of osmium nanoparticles was below 10 nm, which also confirms the results from the transmission electron microscopy (TEM) (Figure 4e and Figure 5e).

### 3.2. Performance of Os–NP/nDol Membrane in the Catalytic Reduction of p–Nitrophenol (pNP) to p–Aminophenol (pAN) with Molecular Hydrogen

The reduction of *p*-nitrophenol, with hydrogen obtained in situ from sodium borohydride (NaBH_4_), was studied to determine the catalytic performance of various nanomaterials [14,15,16,37,38,39,40,45].

**Figure 10:**
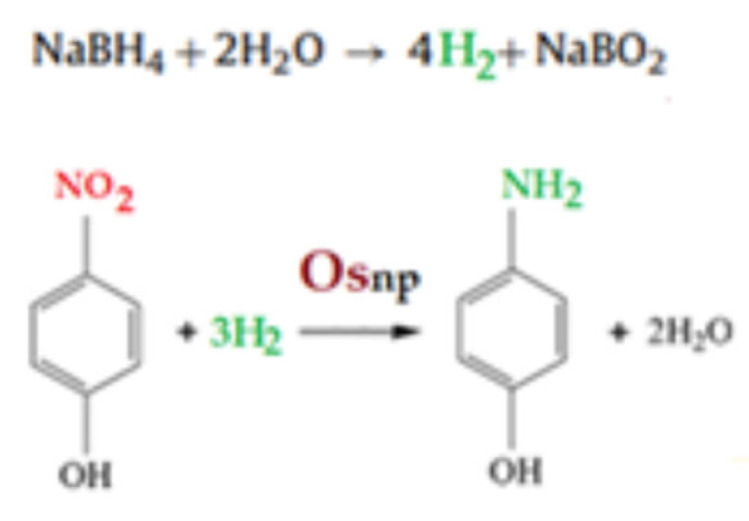
Reaction schematic for the catalytic reduction of *p*–nitrophenol (*p*NP) to *p*–aminophenol (pAN) with molecular hydrogen.

Schematically (Figure 10), the reduction reaction takes place after the formation of molecular hydrogen through the intervention of osmium nanoparticles [14,15,16].

In the study, the conversion of *p*–nitrophenol (*p*NP) to *p*–aminophenol (*p*AN) was determined by varying the concentration of nanoparticles in the membrane nanodispersion, but also the flow rate of the source phase through the membrane and the efficiency of the separation of *p*–aminophenol (*p*AN), formed depending on the membrane flow rate through the variable pH of the receiving phase.

#### 3.2.1. The Conversion of *p*–Nitrophenol (pNP) to *p*–Aminophenol (pAN)

The source phase containing a *p*-nitrophenol substrate with a concentration of 2.0 g/L and 5 g/L sodium borohydride (1000 mL) is passed in the form of free-falling drops through the stationary nanodispersion (500 mL). In the present case, the catalytic effect is obtained by making contact between the variable flow rate (20–120 mL/min) source phase and the membrane nanodispersion (Figure 3). Periodically, the concentration of *p*–nitrophenol is determined spectrophotometrically, in order to compute the conversion yield.

The conversion efficiency (η%) of *p*–nitrophenol to *p*–aminophenol is calculated either in terms of concentration (3) or using the absorbance of the samples (4) [45,46,47].

**Figure 11:**
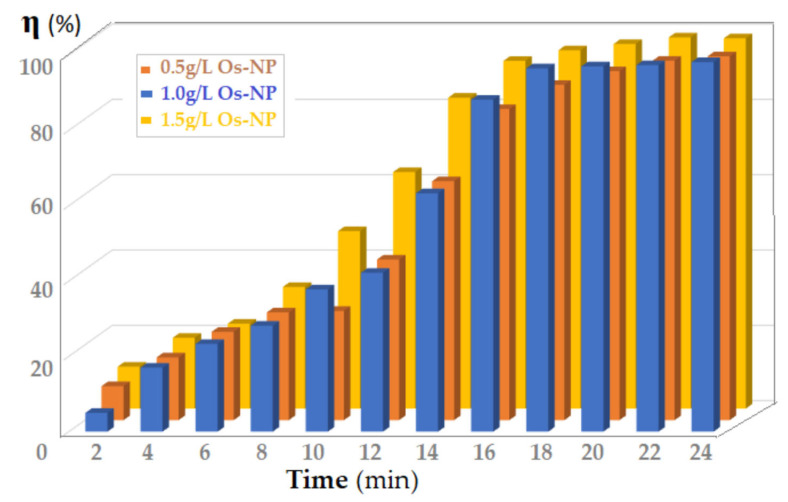
The variation in *p*–nitrophenol conversion (η), depending on the flow rate of the source phase (1000 mL) and the contact time with the membrane phase containing either 0.5, 1.0 or 1.5 g/L osmium nanoparticles (Os–NP).

Figure 11 shows the results obtained for the conversion (η) of *p*–nitrophenol by making contact between the source phase (1000 mL) and the membrane phase, containing either 0.5, 1.0 or 1.5 g/L osmium nanoparticles (Os–NP). The source phase flow rate through the membrane nanodispersion was 100 mL/min. Although the conversion yield increased with the increase in Os–NP concentration in the membrane nanodispersion, the variation of the catalytic effect was small, which suggests that the process is dependent on the contact surface.

**Figure 12:**
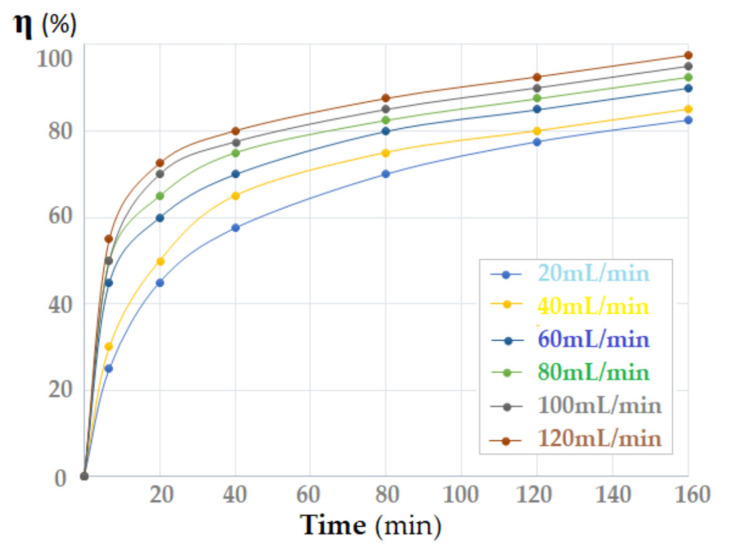
The variation in *p*–nitrophenol conversion (η) depending on the flow rate of the source phase (1000 mL) and the contact time with the membrane phase, containing 1.0 g/L osmium nanoparticles (Os–NP).

To verify this hypothesis, the flow rate of the source phase was varied between 20 and 120 mL/min (Figure 12). The source phase, passing into droplets through the membrane dispersion, has a contact surface depending on the flow rate (which influences the size of the droplets) [46,47].

The conversion (η) of *p*–nitrophenol increased with the increase in the flow rate of the source phase through the membrane. In the first part of the contact time, an important variation in the conversion depending on the flow was observed, but after one hour of contact this variation faded.

After the tests, it was established (Table 1) that the value of the apparent catalytic constant (*k_app_*) was close to the results known from the specialized literature [14,46,47,48].

The data in Table 1 were calculated similarly to the comparison data, taking into account the most probable kinetic equation (5) [49,50]:

(5) $$ln\;\left(C/C_{0}\;\right)=-k\cdot K\cdot t=-k_{app}\cdot t$$

with *C* being the concentration of the reactant (mg/L), *t* being the reaction time, *k* being the reaction rate constant (mg/(L×min)), *K* being the adsorption coefficient of the reactant (L/mg), and *k_app_* being the apparent catalytic rate constant when the concentration (*C_0_*) is very low.

The reaction kinetics describe the *p*NP reduction as a reaction of a pseudo first-order reaction.

#### 3.2.2. Efficiency of Separation of *p*–Aminophenol (*p*AN) Obtained by Reduction of *p*–Nitrophenol (*p*NP) 

**Figure 13:**
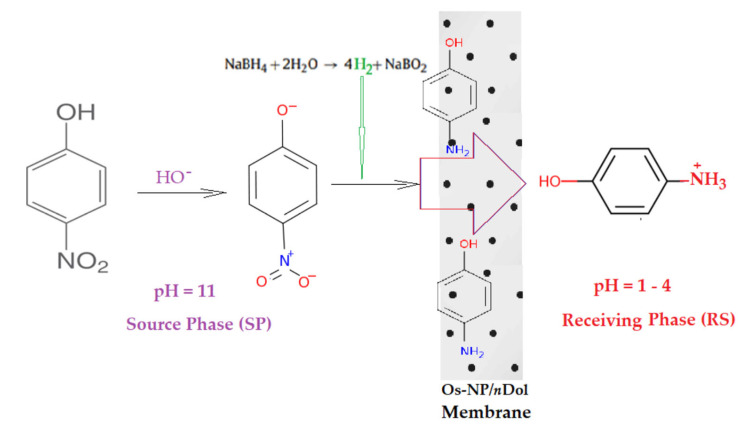
Schematic presentation of the reduction process of *p*–nitrophenol (*p*NP) and transport of *p*–aminophenol (*p*AN) obtained catalytically on osmium nanoparticles in *n*–decanol (OsNP/*n*Dol).

The proposed reaction process aimed to eliminate *p*–nitrophenol (*p*NP) from the source phase by catalytic reduction, but the actual membrane process ended with the recovery of *p*–aminophenol (*p*AN) formed by immobilization in the receiving phase (Figure 3 and Figure 13). In the membrane transportation study, a 10 L source phase was used containing 2.0 and 5 g/L sodium borohydride, 500 mL membrane dispersion with 1.0 g/L osmium nanoparticles and 1L of acid for the receiving phase (HCl), of concentration 10^−4^–10^−1^ mol/L. The flow rate of the source phase was set to 100 mL/min and the membrane flow rate through the stationary receiving phase varied from 5 to 50 mL/min.

**Figure 14:**
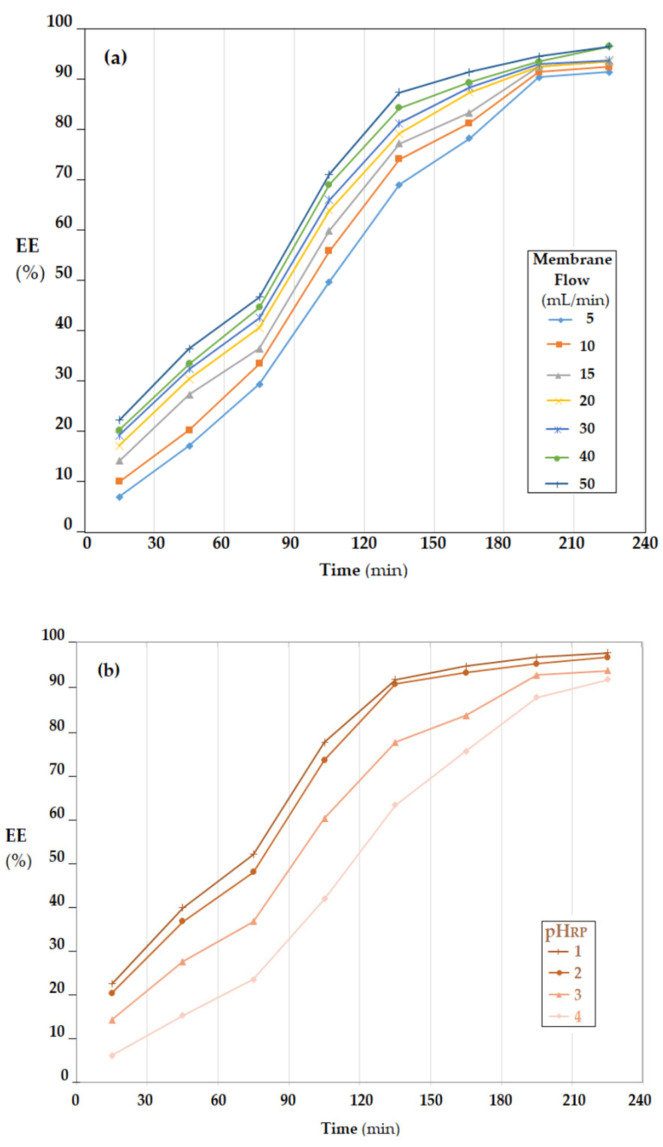
Extraction efficiency (EE) of *p*-nitrophenol (*p*NP) depending on: (**a**) the membrane flow through the receiving phase; and (**b**) pH of the receiving phase.

The efficiency of *p*–aminophenol transport and collection in the receiving phase depended on the membrane nanodispersion flow rate through the receiving phase (Figure 14a) and the pH of the receiving phase (Figure 14b).

The increase in the flow rate of the membrane dispersion through the receiving phase led to the increase in the mass transfer surface and convective transport, thus having a favorable effect on the extraction (Figure 14a). However, the flow rate could not be increased unchecked because secondary emulsification or coalescence phenomena could have occurred [51,52]. For this reason, the flow rate of 40 mL/min was chosen in the experiments regarding the effect of the receiving-phase pH.

The value of source-phase (SP) pH, being conditioned by the amount of 5 g/L sodium borohydride, was strongly alkaline during the process of transporting the *p*–aminophenol (*p*AN) formed to the receiving phase (RP). To ensure a pH gradient favorable to the extraction of *p*–aminophenol (*p*AN) formed in the receiving phase, an acidic pH was required. The lower the pH value, the higher the extraction efficiency (EE) throughout the process. To streamline and optimize the process, it must be taken into account that a low pH means additional hydrochloric acid consumption, and if the pH value increases, the operating time must also increase (Figure 14b). A technically and economically acceptable pH should be set at around 2.

## 4. Conclusions

This paper presents the obtainment and characterization of a liquid membrane based on osmium nanoparticles obtained directly in *n*–decanol (*n*Dol), which is the basic solvent of the liquid membrane.

The dispersion of osmium nanoparticles in *n*–decanol was carried out by the method of reducing osmium tetroxide with 1–undecenoic acid (UDA). The resulting membrane was characterized by transmission electron microscopy (TEM), scanning electron microscopy (SEM), energy-dispersive spectroscopy analysis (EDAX), thermoanalysis (TG, DSC), Fourier transform infra-red (FTIR) spectroscopy and dynamic light scattering (DLS).

In this study, the conversion of *p*-nitrophenol (*p*NP) to *p*–aminophenol (*p*AN) was determined by varying the concentration of nanoparticles in the membrane nanodispersion, but also by varying the flow rate of the source phase through the membrane and the efficiency of the separation of the *p*–aminophenol (*p*AN) formed, depending on the membrane flow rate through the variable-pH receiving phase.

Considering the reaction kinetics describing the reduction of *p*NP as a pseudo-first-order reaction, the apparent catalysis constant lies in the range 0.8 × 10^−4^–4.9 × 10^−4^ (mmol·s^−1^).

The obtained results show that the membranes based on the dispersion of osmium nanoparticles in n-decanol can be used with an efficiency of over 90% for the reduction of *p*–nitrophenol and the separation of *p*–aminophenol.

## Figures and Tables

**Table 1:** Comparative data of the ‘apparent catalytic rate constant (*k_app_*)’ for the catalytic reduction processes.

Catalytic Material	k_app_ (s^−1^)	Reference
Os–polypropylene hollow fiber	1.01 × 10^−4^–8.05 × 10^−4^	[14]
Nanofibers PtNi/SiO_2_	434 × 10^−3^	[46]
Nanofibers Ni/SiO_2_	18 × 10^−3^	
Nanofibers Pt/SiO_2_	55 × 10^−3^	
Ni–Ca–Al_2_O_3_	2.85 × 10^−3^	[47]
Ni catalysts	1.02 × 10^−3^	
Ni–Al_2_O_3_	1.42 × 10^−3^	
Nanofibers Ni–P 0.25/NFM 4.55	18.04 × 10^−3^–26.84 × 10^−3^	[48]
Os–NP/*n*-decanol	0.8 × 10^−4^–4.9 × 10^−4^	This study

## Data Availability

Not applicable.
